# Micropatterned Fibrous Scaffold Produced by Using Template-Assisted Electrospinning Technique for Wound Healing Application

**DOI:** 10.3390/polym13162821

**Published:** 2021-08-22

**Authors:** Norul Ashikin Norzain, Zhi-Wei Yu, Wei-Chih Lin, Hsing-Hao Su

**Affiliations:** 1 Department of Mechanical and Electro-Mechanical Engineering, National Sun Yat-Sen University, Kaohsiung 80424, Taiwan; d063020008@student.nsysu.edu.tw (N.A.N.); m073020065@student.nsysu.edu.tw (Z.-W.Y.); 2 Department of Otorhinolaryngology, Head and Neck Surgery, Kaohsiung Veterans General Hospital, Kaohsiung 813, Taiwan; shsu@vghks.gov.tw

**Keywords:** electrospinning, template-assisted, patterned nanofibers, tissue engineering

## Abstract

This paper describes the fabrication of a structural scaffold consisting of both randomly oriented nanofibers and triangular prism patterns on the scaffold surface using a combination technique of electrospinning and collector templates. The polycaprolactone (PCL) nanofibers were electrospun over a triangular prism pattern mold, which acted as a template. The deposited scaffold was removed from the template to produce a standalone structural scaffold of three-dimensional micropatterned nanofibers. The fabricated structural scaffold was compared with flat randomly oriented nanofibers based on in vitro and in vivo studies. The in vitro study indicated that the structural scaffold demonstrated higher fibroblast cell proliferation, cell elongation with a 13.48 ± 2.73 aspect ratio and 70% fibroblast cell orientation compared with flat random nanofibers. Among the treatment groups, the structural scaffold escalated the wound closure to 92.17% on day 14. Histological staining of the healed wound area demonstrated that the structural scaffold exhibited advanced epithelization of the epidermal layer accompanied by mild inflammation. The proliferated fibroblast cells and collagen fibers in the structural scaffold appeared denser and arranged more horizontally. These results determined the potential of micropatterned scaffolds for stimulating cell behavior and their application for wound healing.

## 1. Introduction

Electrospinning is an efficient fabrication technique used to produce continuous micro- to nanoscale fibers from various natural and synthetic polymer solutions. As the name implies, the driving force of this technique is electrostatic repulsion, wherein a pendant drop of polymer at the tip of a needle is highly electrified by employing the strong electrostatic force between the needle tip and the collector [1,2,3,4]. When the electrostatic repulsion force is exerted and overcomes the surface tension of the polymer solution, the polymer solution is discharged from the needle tip. The electrified polymer jet then undergoes a stretching and whipping process, leading to the production of one-dimensional fibers [1,5]. As the polymer jet is constantly stretched out, the solvent is evaporated and, finally, it is deposited on the assigned collector. The fibers produced from electrospinning have attracted considerable research interest as an application for tissue engineering. This is mainly due to their dimensions being comparable to the native extracellular matrix (ECM) and being excellent substrates that support cell adhesion and proliferation [6]. However, fibers produced by conventional electrospinning are non-woven and randomly oriented, which is different from the topographical ECM [3,7]. It is known that specific topographies can enhance the biological response; hence, there is significant interest in controlling the spatial arrangement of electrospun fibers to produce patterned structures [4,7,8].

To date, numerous approaches have been developed to produce electrospun fibers with different structures [4,8,9,10,11,12]. They can be collected as aligned oriented fibers by using modified collectors of different forms and arrangements, such as rotating drums [13], separate electrodes and metal rings [14,15], or using external electric and magnetic fields [8,16]. Aligned nanofibers can provide contact guidance for the cells, resulting in elongation, cells being aligned along the axes of fibers and faster migration compared to a random orientation [3,7,17]. On top of that, template collectors such as grid-like structures, woven wire mesh and regular hexagons have been used to produce different pattern designs [9,10,11]. Kakunuri et al. produced three-dimensional fibrous micropatterns by using nylon mesh as a collector template and the resultant fibers were more advantageous to the tunable wettability scaffold [9]. Meanwhile, Shin et al. applied femtosecond laser-assisted patterning to fabricated microgroove-patterned nanofibers and demonstrated that microgroove nanofibers promoted the orientation and elongation behavior of endothelial cells [12]. Although patterned electrospun nanofibers have been effectively engineered by these approaches, they are restricted in versatility, requiring a certain electrode design and a complicated system.

Inspired by the advantages of patterned electrospun nanofibers and micropatterning that could stimulate the biological response, we fabricated structural nanofibrous scaffolds to induce fibroblast cell alignment and investigated the viability of fibroblast cells. The structural scaffold consisted of both random fibers and a defined three-dimensional microtopography at the top of the scaffold surface using a combination of electrospinning and collector templates. A triangular prism pattern mold as the collector template was used in this study, inspired by Razali et al.; they demonstrated that a triangular micropatterned film significantly regulated myoblast cell alignment and promoted elongation behavior [18]. The structural scaffold was compared with randomly oriented nanofibers, based on in vitro and in vivo studies. In particular, the structural scaffold was applied to treat the full-thickness wound of rats to investigate the impact of the structural scaffold on the wound closure rate. Finally, the regenerating skin tissue was evaluated through histological staining analysis.

## 2. Materials and Methods

### 2.1. Fabrication of the Polycaprolactone (PCL) Scaffolds

**Figure 1:**
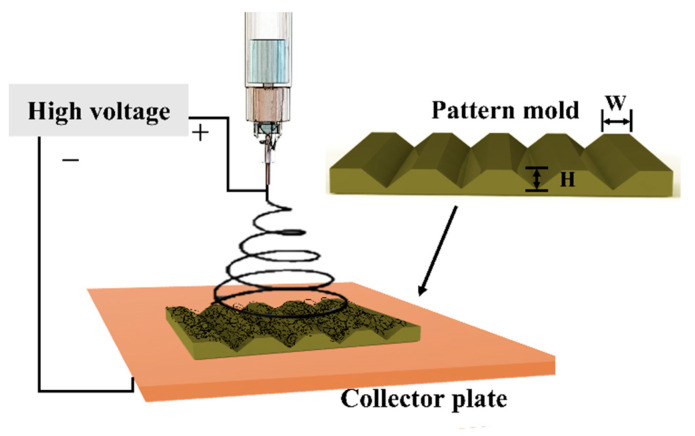
The electrospinning process for fabricating structural scaffold. The alphabet ‘H’ and ‘W’ represents the height of the triangular prism and width between each of the triangular prism, respectively.

The nanofibrous scaffolds were fabricated using electrospinning techniques with the optimal parameters according to our experimental results [19,20,21]. The initial process started by dissolving the polycaprolactone (PCL) granule [19,21,22] into the solvent of 1,1,1,3,3,3-hexafluoro-2-propanol (HFIP, 99%, Sigma-Aldrich, Kaohsiung, Taiwan) for making 20 wt% polymer concentrations. The PCL solution was mechanically stirred overnight until the polymer solution became homogenous. For general electrospinning, the PCL solution was carefully transferred into a commercial plastic syringe equipped with a 23G stainless steel needle. The commercial plastic syringe was attached to the syringe pump that deposited the PCL solution at 0.45 mL/h. Subsequently, alligator clips were used to connect the positive electrode of a high voltage power supply (Nanon-01A, MECC Co., Ltd., Fukuoka, Japan) to the needle tip and the negative electrode was attached to the collector plate. A 15 kV voltage was applied to the needle tip and a 160 mm distance between the needle tip and the collector was maintained throughout the electrospinning process. The PCL flat nanofibers, referred to as two-dimensional (2D) scaffolds, were collected at the aluminum foil. Prior to fabricating PCL structural scaffold, a pattern silicon mold was manufactured through a standard photolithography process. The pattern silicon mold consists of a triangular prism microstructure, where the height (H) of the triangular prism and width (W) between each of the triangular prism were 70 µm and 10 µm, respectively. Subsequently, the pattern mold was used as a collector plate for transferring the triangular prism pattern onto the scaffold surface in order to fabricate the PCL structural scaffold. The PCL nanofibers were deposited onto the pattern mold and simultaneously replicated the pattern of the mold. To produce a thicker scaffold, the 2D scaffold was collected after 1 h electrospun and the structural scaffold was collected after 2 h. The PCL structural scaffold was separated from the pattern mold and samples were vacuum dried overnight to eradicate the residual solvent before further characterization. Figure 1 illustrates the electrospinning setup for fabricating PCL structural scaffold.

### 2.2. Characterization of the PCL Scaffolds

The surface topography of the PCL structural scaffold was observed under a scanning electron microscope (SEM; SU-8000, Hitachi, Tokyo, Japan). The PCL structural scaffold was peeled off from the pattern mold, cut into 10 mm × 10 mm samples and attached to the SEM stub with carbon tape. Each of the samples was sputter-coated for 100 s with a thin layer of titanium to reduce the charging effect during the observation process. The coated samples were then observed under the high vacuum mode of the SEM at 10 kV accelerating voltage. Afterward, the SEM images were imported into ImageJ (National Institute of Health, Bethesda, MD, USA) to measure the fiber diameters, fiber orientations and pattern size of the PCL structural scaffold. The fiber orientations of the structural scaffold were determined by the angle between the fibers and pattern direction. The average fiber diameters of samples were calculated over 100 fibers from randomly selected samples. A similar process and protocol were performed for observing the surface morphology of the PCL 2D scaffold.

The mechanical properties of fabricated scaffolds were determined using a tensile testing machine (FGS-50E-H, Nidec-Shimpo Corporation, Kyoto, Japan). The fabricated scaffolds were detached, cut into a rectangle shape and vertically attached to the machine for testing. The mechanical properties of the fabricated scaffolds were analyzed in both the parallel and perpendicular directions relative to the pattern surface. This was to investigate the effect of the pattern on scaffold strength. The samples were stretched at a speed of less than 20 mm/min until failure. The Young’s modulus, tensile strength and tensile strain were determined from the data obtained.

Numerous research findings have verified that the wettability of polymer nanofibers could significantly affect tissue regeneration [19]. The wettability of the PCL 2D and structural scaffolds was examined using a contact angle instrument (DSA 1000, KRÜSS GmbH, Hamburg, Germany). The fabricated scaffolds were placed on a glass slide and then 5 µL of ultrapure water was dropped onto the sample surface. The water contact angle of the samples was analyzed in both the parallel and perpendicular directions relative to the pattern surface. Images of the water droplet were captured and further processed to obtain the value of the contact angle. The contact angle value was calculated as the average of 15 measurements.

### 2.3. In Vitro Study of the PCL Scaffolds

The fabricated scaffolds were evaluated by biological characterization to explicate their influence on adult human dermal fibroblast (HDF) cell behavior. The adult HDF cell line was derived from ATCC (PCS-201-012) and purchased from Bioresource Collection and Research Center (BCRC, Hsinchu, Taiwan). Before the seeding procedure, the fabricated scaffolds were fixed in a 24-well plate and sterilized by UV light for 1 h. The process was initiated by incubating the HDF cells at 37 °C in Dulbecco’s modified Eagle medium (DMEM) supplemented with 10% fetal bovine serum (FBS) and 1% penicillin-streptomycin and antibiotic-antimycotic in a humidified atmosphere of 5% CO_2_. The medium was changed daily and subcultured in a tissue culture plate (TCP) until it reached 90% confluency. At this point, cells were detached using trypsin-EDTA and centrifuged at 1300 rpm for 5 min. The supernatant was removed and a fresh culture medium was added. Subsequently, 1.0 mL of cell medium suspension was seeded on the surface of the fabricated scaffold at a density of 4 × 10^4^ cells per well and incubated in the standard conditions of 5% CO_2_ at 37 °C. The HDF viability study was performed for 8 days using a Cell Counting Kit-8 assay (CCK-8, Dojindo, Kumamoto, Japan). After the elapsed time, the culture medium was removed and the samples were washed with phosphate-buffered saline (PBS) three times. Then, a 0.5 mL medium consisting of 10% CCK-8 solution was added into each well and incubated for 2 h. 100 µL of the solution was pipetted into a 96-well plate and subsequently the value of the optical density (OD) of each well was determined at 450 nm absorbance using a microplate reader (Bio-Rad model 680, Genmall Biotechnology Co., Hercules, CA, USA).

A fluorescence microscope and SEM were employed to observe the morphologies and distribution of the HDF cells on the fabricated scaffolds. First, the nucleus of cells was prestained with 4 mL of 4′,6-diamidino-2-phenylindole (DAPI, Sigma–Aldrich, Kaohsiung, Taiwan) staining solution, incubated overnight and excessive DAPI staining solution was washed away with PBS three times. For the cell body staining, the cells were stained with 0.5 mL of fluorescein diacetate (FDA, Sigma–Aldrich, Kaohsiung, Taiwan) staining solution, incubated for 5 min and then rinsed three times to remove the excessive FDA staining solution. The fluorescence of cells was observed and digitally recorded using a microscope with NIS-Elements AR software (Eclipse Ni-U, Nikon, Tokyo, Japan). All the processes of cell fluorescence staining were conducted on days 3 and 8 in a dark environment at room temperature.

**Figure 2:**
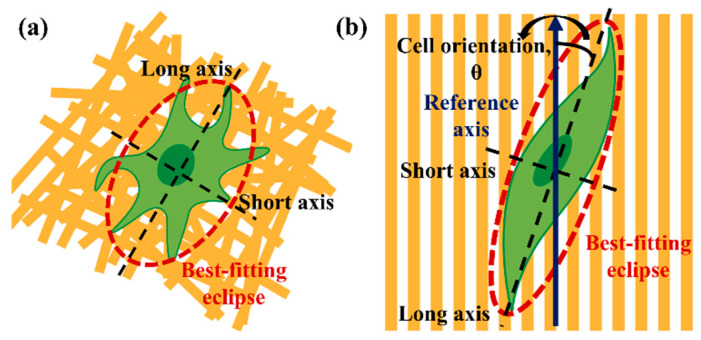
The evaluation of cell elongation and orientation. Illustration of (**a**) cell on the tissue culture plate or flat random scaffold and (**b**) cell on the structural scaffold. Cell elongation in terms of aspect ratio was measured by dividing the length of the long axis by the short axis. The cell orientation was determined by the angle between the long axis of the ellipse and the pattern direction.

To investigate the effect of cell behavior on the fabricated scaffolds, the elongation and orientation of HDF cells were determined by analyzing the photographed fluorescence of cells. A color threshold in the ImageJ software was used to automatically segment the fluorescent cells and trace the best-fit ellipse of the cells. From the cell’s best-fit ellipse, the length of the long and short axes was determined, as shown in Figure 2. Then, the cell elongation in terms of aspect ratio was measured by dividing the length of the long axis by the short axis. The cell orientation was determined by the angle between the long axis of the ellipse and the pattern direction. The pattern direction was referred as references axis. The cell orientation (θ) was ranged from 0° to 180°. The cells were considered to have arbitrary orientation when the angle (θ) exceed 10°. The average aspect ratio and cell orientation were determined from 40 nuclei.

For morphological observation using SEM, the samples were washed with PBS to remove the floating cells, fixed with 0.5 mL of 2.5% glutaraldehyde (GA, Sigma-Aldrich, Kaohsiung, Taiwan) for 2 h and then washed again three times with PBS. The fixed samples were sequentially dehydrated with ethanol concentrations of 30%, 50%, 75%, 90%, 95%, 99.5% and 99.5%. Finally, SEM imaging of fully dried samples was performed.

### 2.4. In Vivo Study of the PCL Scaffolds

All animals were treated and used according to the protocol under approval no. 10738 by the Animal Care and Use Committee of National Sun Yat-sen University. All efforts were made to minimize animal discomfort and minimize the number of animals used for the experiment. Twelve healthy male Wistar rats (BioLASCO Taiwan Co., Taipei, Taiwan) weighing 276–300 g (8 weeks old) were utilized in this experiment. The rats were anesthetized with 2.5% isoflurane and the dorsal region of the rats were shaved and disinfected with 70% ethanol. Then, two 15 mm circular diameter full-thickness excisional wounds were created side by side. The injured rats were randomly distributed into four different groups (*n* = 3 for each group) of no treatment, commercial gauze, PCL 2D scaffold and PCL structural scaffold. All of the fabricated scaffolds were cut into 2 cm × 2 cm samples and sterilized with UV light for 1 h before the treatment. Subsequently, the wound region was treated with the assigned scaffold. For the application of structural scaffold, the top surface of the sterilized structural scaffold was placed directly in contact with the full-thickness wound and then fixed with surgical tape. This purpose is to keep the scaffold position to the wound site. In addition to that, the assigned scaffolds were changed every 2 days, particularly the structural scaffold for maintaining the triangular prism microstructure effect to the wound area. The rats were placed in a cage and fed individually. The wound area was evaluated for all treatment groups on days 0, 4, 7 and 14 by imaging the wound area. The wound healing percentage was calculated by using the following equation:

$$\mathrm{Wound}\;\mathrm{healing}\;\mathrm{percentage}\;=\;\left(\frac{A_{i}-A_{t}}{A_{i}}\right)\;\times \;100\%$$

where $A_{i}$ and $A_{t}$ are the initial wound area and wound area at time of measurement, respectively.

### 2.5. Histological Analysis

The healed skin tissue of the healed wound area of Wistar rats was analyzed using histological staining. The rats in each group (*n* = 3) were anesthetized and sacrificed on day 14 post-surgery. The skin tissue samples were collected by excising the full thickness of the wound together and fixed in 4% paraformaldehyde (Sigma-Aldrich, Kaohsiung, Taiwan) at 4 °C. Subsequently, the skin tissue samples were embedded in paraffin and cut into sections 5 µm thick. Finally, the section samples were stained with hematoxylin and eosin (H & E). The section samples were imaged using a digital slide scanner (MoticEasyScan, Motic, Xiamen, China) for histologic analysis and scored using the International Harmonization of Nomenclature and Diagnostic Criteria (INHAND) protocol [23,24]. The re-epithelization was graded as 0 for absence, 1 for scant presence, 2 for moderate presence and 3 for remarkable presence, while the orientation of collagen fibers and fibroblasts was graded as 0 for absence, 1 for vertical arrangement, 2 for mixed arrangement and 3 for horizontal arrangement. Inflammation was also scored as 0 for no remarkable (<1%), 1 for minimal (1–5%), 2 for slight (5–25%), 3 for moderate (26–50%), 4 for moderately severe (51–75%) and 5 for severe (>75%). Finally, the average data was reported.

### 2.6. Statistical Analysis

All the experiment data were expressed in mean ± standard deviation (mean ± SD). Statistical difference between the groups was determined using one-way analysis of variance (ANOVA) with Turkey’s post-test. The findings were considered low statistical different when the *p*-value was <0.05 (*), moderate statistical different when the *p*-values was <0.01 (#) and high statistical different when the *p*-value was <0.001 (&).

## 3. Results and Discussion

### 3.1. Characterization of the Fabricated PCL Scaffolds

**Figure 3:**
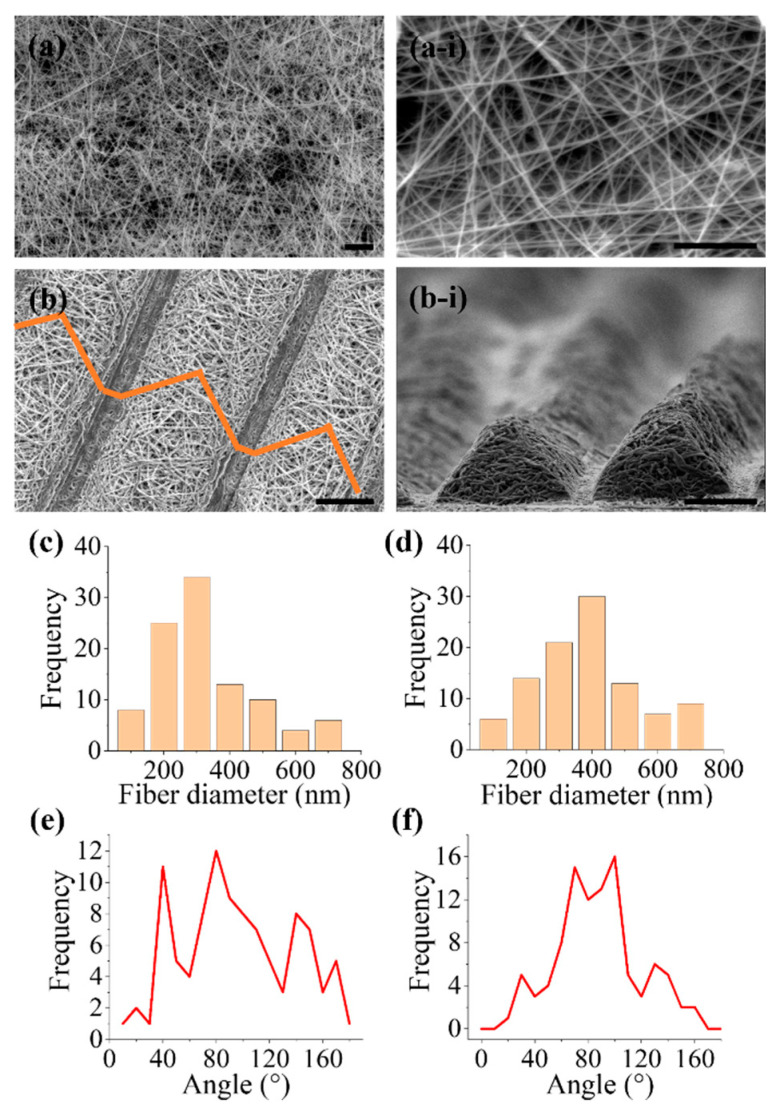
Scanning electron microscope (SEM) images of (**a**,**a-i**) 2D scaffold in random orientation, (**b**) structural scaffold at the top view and (**b-i**) structural scaffold at the side view. Fiber diameter distribution for (**c**) 2D scaffold and (**d**) structural scaffold. Fiber orientation distribution for (**e**) 2D scaffold and (**f**) structural scaffold. The scale bar for (**a**,**a-i**) represents 10 µm and 5 µm, respectively. The scale bar for both (**b**) and (**b-i**) represents 50 µm.

In the present study, two nanofibrous scaffolds with significantly different morphologies were fabricated by modifying the collector template used for electrospinning. A 2D scaffold with a random orientation was collected at the plate collector covered with aluminum foil. A pattern as a collector template was used to fabricate a structural scaffold, which consisted of both random nanofibers and triangular prism patterns on the surface of the scaffold. The morphologies of different PCL scaffolds were observed at high resolution using an SEM, as demonstrated in Figure 3. Known electrospinning techniques will produce randomly oriented fibers and the quantitative analysis of SEM images showed that the distribution of the fiber diameters of the 2D scaffold ranged between 100 nm to 700 nm. The fibers were randomly oriented ranging from 0–180° angle interval. Figure 3b presents the structural scaffold fabricated using a combination of electrospinning and pattern as a collector plate. From the SEM observation, the fabricated triangular topography was successfully fabricated on the surface of a random nanofiber scaffold. The resultant structural scaffold demonstrated an organized topographical structure with dense random nanofibers. Cross-sectional SEM images showed that by electrospinning the PCL nanofibers onto the pattern mold, the combination of techniques was able to reproduce the outline of the mold on the scaffold surface. Similarly, the structural scaffold consisted of fibers with diameters ranging between 100 nm to 700 nm. This is expected from electrospinning approach, where the nanofiber produced in a wide range of nanoscale and less uniformity [3]. The structural scaffold consists of 57% nanofibers distributed at 70–100° angle intervals respective to the pattern direction. This indicated there is slight alignment might occur in the structural scaffold. The height of the triangular prisms was 68.35 ± 21 µm and the distance between each of the triangular prisms was 14.29 ± 34 µm, while the average fiber diameter for the 2D scaffolds was 329 ± 186 nm and the average fiber diameter for the structural scaffolds was 414 ± 164 nm.

The mechanical properties of fabricated PCL scaffolds were determined by analyzing the data obtained from testing. From the analysis, 2D scaffolds presented consistent mechanical properties in terms of tensile strength, tensile strain and Young’s modulus as shown in Table 1, irrespective of orientation [3]. The mechanical properties of the structural scaffolds also demonstrated a similar trend to the 2D scaffold, wherein there was no difference regardless of orientation. From the analysis, the micropattern did not significantly affect the scaffold strength.

**Figure 4:**
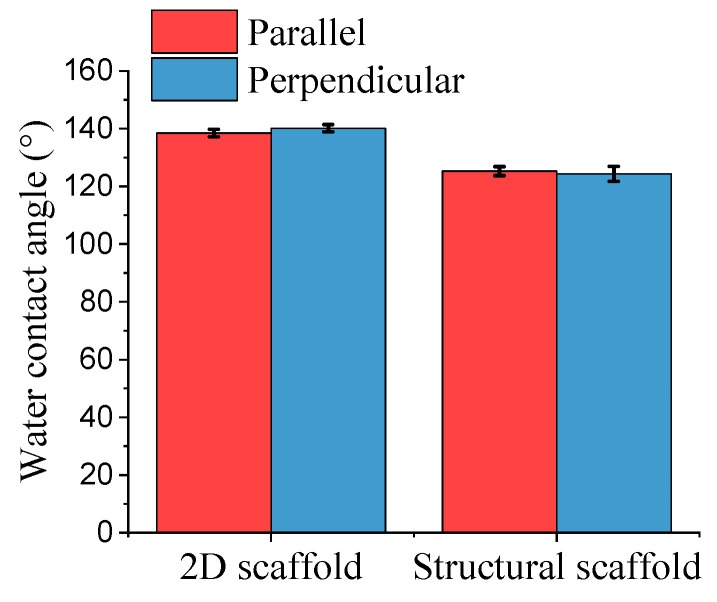
Water contact angles of different morphologies of polycaprolactone (PCL) scaffolds.

The wettability of the fabricated PCL scaffolds was determined by analyzing the water contact angle. The water contact angle of the fabricated scaffolds was measured in parallel and perpendicular directions. Figure 4 shows the mean water contact angle of the fabricated PCL scaffolds. From the analysis, all the fabricated scaffolds had a water contact angle of over 100°, demonstrating that all the samples were hydrophobic. The obtained results are consistent with previous reports on randomly oriented nanofibers [12,25]. The 2D scaffold had a similar water contact angle in both parallel and perpendicular directions. The water contact angle for the 2D scaffold in parallel and perpendicular directions were 138.47 ± 1.25° and 140.14 ± 1.23°, respectively. The water contact angle for the structural scaffold in parallel and perpendicular directions was slightly different, at 125.29 ± 1.58° and 124.33 ± 2.59°, respectively. The results obtained match those of Shin et al., which demonstrated that the wettability of random nanofibers was reduced after a microgroove pattern was fabricated on the nanofiber surface [12]. The water contact angle of microgroove-patterned nanofibers was comparable to aligned nanofibers [12].

### 3.2. HDF Cell Behavior on Different Morphologies of PCL Scaffolds

**Figure 5:**
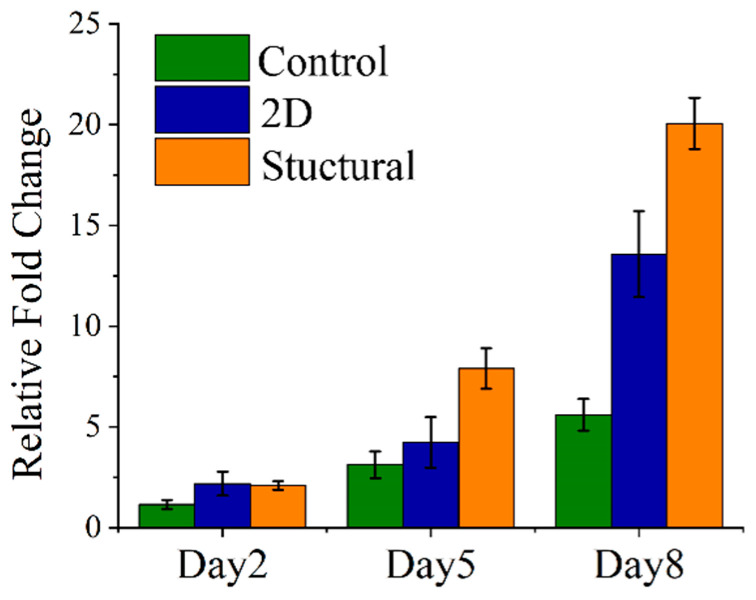
Viability of adult human dermal fibroblast (HDF) cells cultured for 8 days on different morphologies of PCL scaffolds.

Topographical patterning has been widely used to control cell adhesive morphology [18,26,27,28,29]. The HDF cells were cultured on the fabricated PCL scaffolds for 8 days to investigate the capabilities of the structural scaffold in promoting cell growth. Figure 5 demonstrates the viability of HDF cells on different morphologies of PCL scaffolds. In this study, tissue culture plate (TCP) was used as a control. The viability of cells for each group was presented in terms of relative fold change from those cultured for 24 h. In the early stage of incubation, the structural scaffold exhibited cell viabilities comparable to the 2D scaffold on day 2. The fold change of HDF cell viabilities rate for 2D scaffold, structural scaffold and TCP control were 2.33, 2.09 and 1.84 times increased from 24 h of culturing. The structural scaffold gradually assisted the proliferation of HDF cells, increasing to 7.89 times on day 5. By increasing the incubation time, the 2D scaffold demonstrated a lower cell viability compared to the structural scaffold and TCP control presented the lowest cell viability among the groups. The structural scaffold exhibited a higher fold change of cell viability rate, increasing on day 8 to 19.99 times from 24 h of culturing. This phenomenon could be attributed to the 2D scaffold having a highly hydrophobic surface, which leads to the cells being hindered.

**Figure 6:**
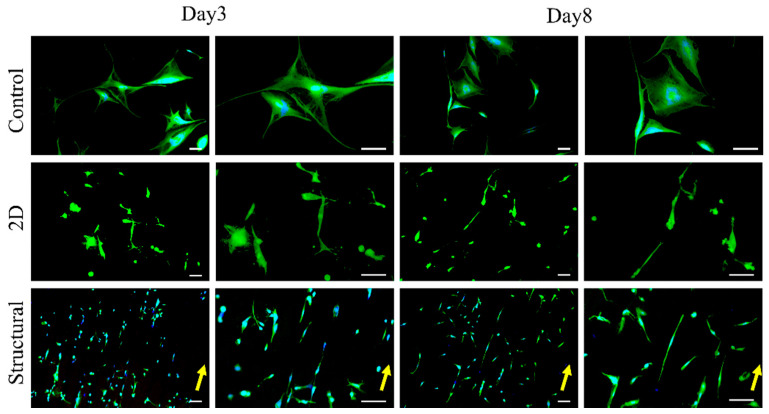
Morphologies of HDF cells on different scaffolds at days 3 and 8. Scale bar represents 100 µm at different magnifications. The yellow arrow indicates the direction of the triangular prism pattern of the structural scaffold.

The HDF cell morphologies on the fabricated scaffolds were observed on days 3 and 8 of incubation. The fibroblast cells adhered to the fabricated PCL scaffolds and the morphologies of cells were expected to be in a spindle shape or dependent on the cultured substrate. Figure 6 presents the HDF cell morphologies on the TCP control and the fabricated PCL scaffolds. From our observation, the HDF cells’ behavior on the 2D and structural scaffold demonstrated significantly different morphologies. The HDF cells on the TCP control were distributed randomly without any preferential direction. Although the cytoskeleton HDF cells stretched along the randomly oriented nanofibers, the cultured cells show no preferential alignment and are distributed arbitrarily. It was noticed that the HDF cells on the structural scaffold exhibited an elongated morphology and aligned along the triangular prism topography on day 3. By increasing the incubation time, the HDF cells favorably moved, migrated and proliferated along the triangular prism of the structural scaffold. The HDF cells on the structural scaffold exhibited a similar elongation and orientation behavior in comparison with cell morphologies on aligned nanofibers or anisotropic topography [3,7,17,29,30,31,32,33].

**Figure 7:**
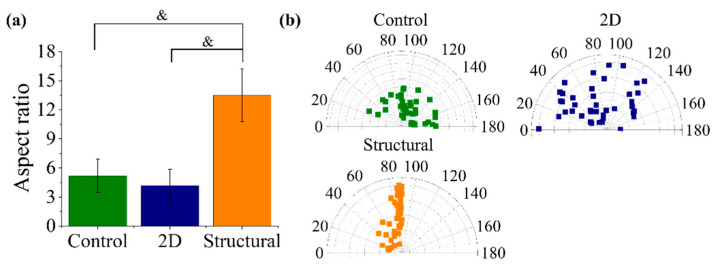
HDF cell alignment in terms of (**a**) aspect ratio and (**b**) orientation after 8 days of incubation on different morphologies of PCL scaffolds. The symbols & represent *p* < 0.001, for comparison between the structural scaffolds to other groups.

A quantitative analysis of the cell elongation in terms of aspect ratio and orientation of cells on the fabricated PCL scaffolds at day 8 is shown in Figure 7a. The structural scaffold changed the stretching of the HDF cells in comparison to the 2D scaffold and TCP control, which correlates with the HDF morphologies in Figure 6. The HDF cells on the structural scaffold exhibited a higher cell elongation with an aspect ratio of 13.48 ± 2.73, while 2D scaffold and TCP control showed moderate cell elongation at day 8 with aspect ratios of 4.16 ± 1.71 and 5.19 ± 1.72, respectively. The angle distribution of HDF cells on the structural scaffold was relatively narrow in comparison to the 2D scaffold and TCP control; 70% of HDF cells aligned along the peak of the triangular prism. This cell orientation results were comparable to the fibers orientation distribution analysis, where approximately 57% nanofibers oriented at 70–100° angle intervals respective to the pattern direction.

**Figure 8:**
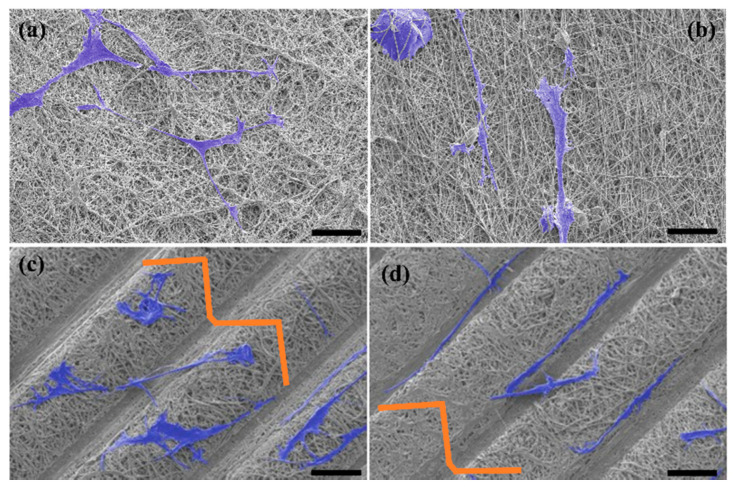
Morphologies of HDF cells on day 8 of incubation on different morphologies of (**a**,**b**) 2D scaffold and (**c**,**d**) structural scaffold. Scale bar represents 50 µm.

This suggests that the triangular prism on the scaffold surface can enhance cell activities in comparison to the 2D scaffold. The results obtained were consistent with previous documentation, wherein cell proliferation on a groove film was higher compared to a flat film [29,33]. Based on the study by Zorlutuna et al., structural properties play an important role in directing cells [29,34]. They reported that triangular microstructural film promoted cell attachment and demonstrated a similar cell behavior to cells cultured on a collagen film [29,34]. In addition, the peak of the triangular prism of the structural scaffold could assist in guiding the fibroblast cells to attach, move and proliferate. The orientation and elongation of fibroblast cells significantly improved when the triangular prism pattern was added to the scaffold surface. Specifically, our findings suggested that nanofibers present a more impact on cell orientation, while the improvement of cell elongation behavior significantly might be enhanced with the aid of triangular prism microstructure. This is supported by studies from Razali et al. and Thery et al., which demonstrated that the peak of the triangular prism has a similar effect to a triangle tip [18,35]. A continuous triangle tip can affect the development of the cell and stimulate lamellipodia and filopodia formation [35]. Figure 8 shows the morphology of HDF cells on the fabricated scaffolds.

Although the fabricated micropattern nanofibers improved the fibroblast cell’s activities which is consistent with previous studies, however, most of the micropattern nanofibers were produced from conventional electrospinning. As known, fibers produced from conventional electrospinning have a less uniform structure, fibers fracture and a wide range of nanofibers [3,36]. These physical and morphological properties could reduce the effect of cell behavior since the cell activities could be decreased with a minimal difference in the fiber diameters [37,38,39,40]. Hence, we assumed a structural scaffold can be fabricated by modified electrospinning [3,36,41,42,43,44] in further studies, which is able to produce fibers with a more uniform distribution. This can lead to provide more positive effects on cell behavior. In addition to that, acknowledged that aligned nanofiber can provide cell contact guidance, we assumed a structural scaffold consisting of aligned nanofibers and triangular prism on the scaffold surface might synergistically enhance the fibroblast cell behavior. However, further studies are needed to clarify the potential phenomena.

### 3.3. In Vivo Study of the Fabricated PCL Scaffolds

**Figure 9:**
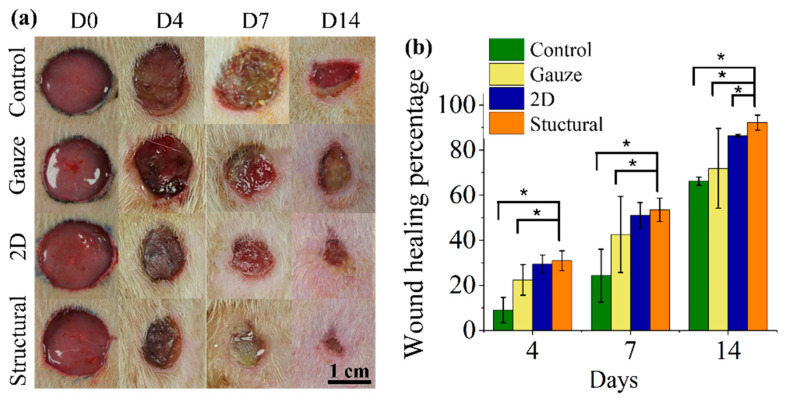
(**a**) Representative images of wounds from different groups: control (no treatment), treated with commercial gauze, treated with 2D nanofibers in random orientation and treated with structural nanofibers at days 0, 4, 7 and 14. (**b**) Analysis of wound healing rate. The symbols * represent *p* < 0.05, for comparison between the structural scaffolds to other groups.

To evaluate the in vivo performance of our structural scaffold, full-thickness skin wounds were created on the dorsal region of rats. The impact of the structural scaffold on the wound region was compared with the 2D scaffold, commercial gauze and no treatment (control) groups. After the surgery, the assigned scaffolds covered the wound site as the dressing for the wounds and were subsequently fixed with a layer of surgical tape to maintain the dressing position. The appearance of each wound was observed on days 0, 4, 7 and 14, as demonstrated in Figure 9a. From photographic observation, treatment by nanofibrous scaffold displayed a faster and better healing process than both the commercial gauze and control groups. During the healing process, the wound area treated with the commercial gauze and 2D scaffold demonstrated a slight redness and swelling. This could be due to the 2D scaffolds having a higher hydrophobicity than the structural scaffold, which hinders the binding of cells. As the treatment time increased, the wound area treated with 2D scaffold and commercial gauze did not significantly decrease. While the diameter of the wound area treated with the structural scaffold displayed an obvious reduction on day 7, the wounds observed in the control and commercial gauze groups demonstrated the lowest reduction in wound size. As observed from the photographs, the diameter of the wound reduced significantly over time, particularly in the structural scaffold group.

At different treatment times, the healing area of wounds was measured based on the macroscopic images in Figure 9a. The wound healing percentages are expressed as the wound size reduction relative to the original area of the wound site, shown in Figure 9b. On day 4, the structural scaffold presented a higher wound healing rate among the treatment groups, achieving 30.8% wound closure. As the healing time increased, the wound area treated by the structural scaffold continuously reduced and 53.49% wound closure was obtained on day 7, whereas the 2D scaffold achieved a 50.94% wound closure on day 7. The commercial gauze and control groups presented the lowest healing rate, achieving a wound closure of 42.58% and 24.26%, respectively. On day 14 of treatment, the wound size in all treatment groups significantly decreased and the healing percentage of the structural scaffold reached 92.17%. The wound area had more complete healing and achieved rapid recovery when the structural scaffold was used as a dressing compared with the 2D scaffold and commercial gauze.

### 3.4. Histological Staining

**Figure 10:**
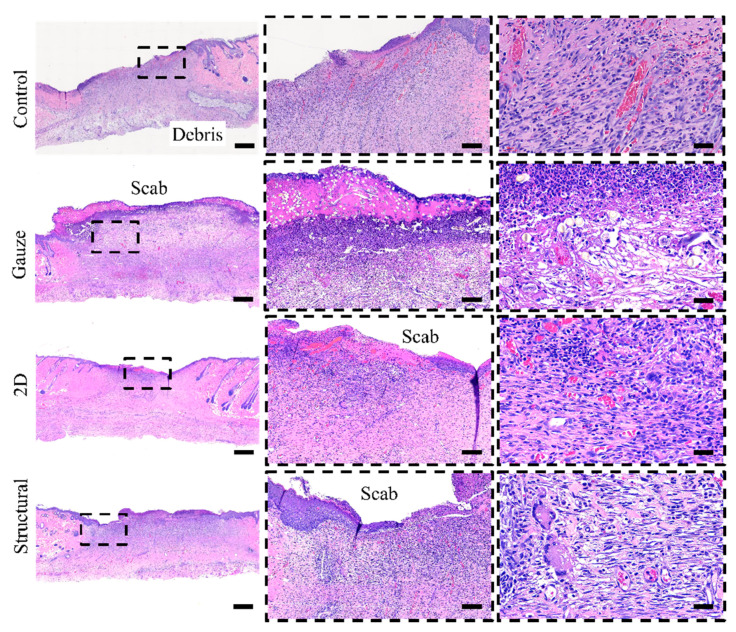
Hematoxylin and eosin (H&E) staining of the healed wound area on day 14. The scale bar represents 600 µm, 100 µm and 30 µm from left to right.

**Figure 11:**
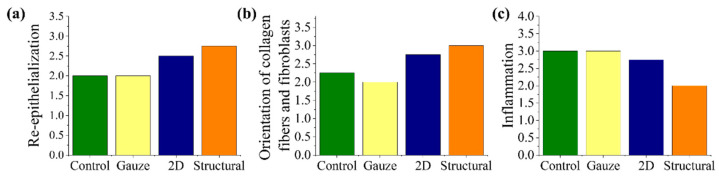
Histological scoring of (**a**) re-epithelization, (**b**) orientation of collagen fibers and fibroblast cells and (**c**) inflammation.

Histological staining was used to evaluate the performance of the structural scaffold during the wound healing process. Reconstruction of the dermis and epidermis layers are key factors in evaluating wound healing and tissue regeneration [45,46]. Re-epithelization starts with the thickening of the epidermis layer near the wound area and also involves the proliferation of fibroblast cells at the dermis layer. The fibroblast cells are responsible for producing connective tissue and an ECM that assists the epidermis in promoting wound closure. Histological analysis of the healed wound area was conducted on day 14 post-surgery, as shown in Figure 10. Based on the histological staining, wounds treated with nanofibrous scaffolds demonstrated better epithelization in comparison to other treatment groups. It was noticed that the collagen fibers in the dermis layer of the 2D and structural scaffold appeared in a more dense and organized form. Most of the collagen fibers and proliferated fibroblast cells are arranged horizontally, particularly in the structural scaffold. The wound areas treated with commercial gauze and no treatment showed a severe delay in re-epithelization with a significant chronic inflammatory stage, as shown in Figure 11. From the staining, a high number of inflammatory cells and vascular formation was observed, particularly surrounding the debris. This is possible because no treatment is an open wound, causing the foreign material in the environment, such as food debris or straw pellet bedding, to become embedded in the wound. The results suggest that the structural scaffold could enhance the wound healing process and tissue regeneration in comparison to the 2D scaffold. The peak of the triangular prism of the structural scaffold could have a positive influence on the arrangement of collagen fibers and proliferated fibroblast cells.

## 4. Conclusions

We have demonstrated the fabrication of two nanofibrous scaffolds with different morphologies by modifying the collector template used for electrospinning and their application in stimulating fibroblast cells. A 2D scaffold with random orientation was collected at the plate collector covered with aluminum foil and a pattern mold as a collector template was used for transferring the triangular prism pattern onto the scaffold surface. Morphological and physical characterization of the structural scaffold revealed that the combination of techniques was able to reproduce and replicate the triangular prism pattern mold. The structural scaffold modulated the adult HDF cells’ alignment and elongation along the triangular prism pattern direction during the incubation time. The in vivo study indicated that the structural scaffold accelerated wound healing closure, achieving 92.17% closure on day 14, while the 2D scaffold reached 86.41% wound closure. The histological analysis revealed that wounds treated with the structural scaffold presented better epithelization on day 14 and the presence of collagen fibers in the dermis layer appeared in a more dense and organized form with only mild inflammation. Most of the collagen fibers and proliferated fibroblast cells are arranged more horizontally in comparison to the 2D scaffold. Our results suggest that engineered micropatterns on the scaffold surface could regulate fibroblast cell behavior, resulting in rapid wound closure throughout treatment.

## Figures and Tables

**Table 1:** Mechanical properties of the fabricated polycaprolactone (PCL) scaffolds.

	Tensile Strength (MPa)		Tensile Strain (%)		Young’s Modulus (MPa)	
Scaffold Direction	Parallel	Perpendicular	Parallel	Perpendicular	Parallel	Perpendicular
2D	2.19 ± 0.06	2.12 ± 0.11	90	81	4.38	4.24
Structural	1.05 ± 0.11	1.12 ± 0.12	26	44	5.01	3.9

## Data Availability

Not applicable.
